# Genetic diversity of ESBL-producing *Escherichia coli* in ICU settings

**DOI:** 10.6026/973206300222724

**Published:** 2026-05-31

**Authors:** Aparna Aparajita, Manoj Kumar, Ashok Kumar Sharma

**Affiliations:** 1 Department of Microbiology, Rajendra Institute of Medical Sciences (RIMS), Ranchi, Jharkhand, India

**Keywords:** Extended-Spectrum β-Lactamase (ESBL), *Escherichia coli*, genetic characterisation, intensive care unit (ICU), CTX-M, multilocus sequence typing

## Abstract

Extended-Spectrum β-Lactamase (ESBL) - producing *Escherichia coli* pose a major threat in ICU settings due to rising multidrug resistance and limited data on their genetic diversity. Therefore, it is of interest to analyze the molecular epidemiology of 186 non- duplicate ESBL-producing *E. coli* isolates from ICU patients over 12 months using Polymerase Chain Reaction (PCR) for ESBL genes, phylogenetic grouping, Multilocus Sequence Typing (MLST) and antimicrobial susceptibility testing. The blaCTX-M gene family was predominant (82.3%), with blaCTX-M-15 as the most common variant and 41.9% of isolates carried multiple ESBL genes. Phylogenetic group B2 and high-risk clones such as ST131, ST405 and ST648 were most prevalent, with high co-resistance to multiple antibiotic classes. Thus, we show significant genetic diversity and dominance of high-risk clones, emphasizing the need for molecular surveillance and targeted infection control strategies.

## Background:

The recent tendency towards the increasing resistance to antimicrobials in Gram-negative bacteria is one of the most difficult challenges of modern healthcare systems across the globe. Of particular concern is the extended-spectrum beta-lactamase (ESBL)-producing *Escherichia coli* that has become a pathogen of strategic importance because of its ability to hydrolyse extended-spectrum cephalosporins and monobactams, effectively rendering these important antimicrobial agents ineffective in treatment [1]. *Enterobacteriaceae* that produce ESBL have been listed as a high priority pathogen and new therapeutic strategies should be developed urgently [2] by the World Health Organization. Intensive care units are epicentres of the development and spread of multidrug-resistant organisms. These factors such as the clustering of critically ill patients with impaired immune defence, high-selective pressure on antibiotics, the use of invasive devices, extended hospitalisation and the presence of frequent patient-to-patient transmission events combine to produce a conducive environment unlike any other in favour of the growth of resistant bacteria [3]. In the ICU setting the ESBL-producing *E. coli* infection is linked to the both high mortality rates, extended hospitalization, increased hospital costs and a reduction in treatment options [4]. Molecular evolution ESBL enzymes have experienced radically different evolutionary changes in the last twenty years. Whereas TEM and SHV forms traditionally predominated in the ESBL epidemiology, the CTX-M family has now gained global prominence in a quicker sequence of horizontal gene transfer, clonal growth and collaboration with highly effective mobile genetic components [5]. The CTX-M-15 type has succeeded in obtaining a pandemic distribution within the CTX-M-family and is now the most common seen ESBL enzyme in the world, specifically in the community-acquired and healthcare-associated *E. coli* infections [6]. Nevertheless, the proportional distribution of various CTX-M groups and their correlation with other resistance determinants are significantly different in different geographic areas and clinical environments [7].

The ESBL producing *E. coli* population structure has shown that there are global disseminated high-risk clonal lineages which act as carriers of resistance genes across different ecological niche. ST131 especially the H30-Rx subclone was found to be the most successful pandemic clone having virulence features, fluoroquinolone resistance and CTX-M-15 genes in one successful single highly transmissible lineage [8]. Other outstanding high-risk clones, such as ST405, ST648, ST38 and ST410, have been reported with an increasing frequency in different healthcare facilities but their role in the dynamics of ICU-specific transmission has not been fully defined [9]. Further understanding of *E. coli* pathogenicity of ESBL-producing strains is also given by phylogenetic classification. The species are conventionally divided into major phylogenetic groups (A, B1, B2 and D, where other groups C, E and F can be recognised using refined classification schemes) each having distinct associations with the presence/absence of virulence factors and clinical presentation [10]. The groups B2 and D in which extraintestinal pathogenic strains of *E. coli* are essentially more likely to harbour virulence factor repertoires than commensal strains of the group mostly belonging to group A and B1 [11]. Since the phylogenetic distribution of ESBL-producing strains in ICU settings is the key to determining the pathogenic burden and the ability to transmit the circulated clones. Although the increasing literature on the epidemiology of ESBL-producing *E. coli* has provided an in-depth analysis of the topic, the studies that combine phenotypic resistance profiling with molecular characterisation of the ESBL gene families, phylogenetic grouping and high-resolution typing in the context of an ICU setting in particular are relatively few [12]. Majority of the current studies have concentrated on either the individual parts of characterisation or have involved a large-scale hospital wide survey not stratified on clinical setting. The selective forces and transmission processes that are specific to the ICUs require special studies to support specific infection prevention and antimicrobial stewardship strategies [13]. Therefore, it is of interest to describe the genetic diversity, molecular epidemiology and clonal distribution of ESBL-producing *Escherichia coli* isolates in ICU settings.

## Materials and Methods:

## Study design and setting:

The study was a cross-sectional observational design; it was carried out during the period of a year (January 2023 to December 2023) in the medical, surgical and neonatal Intensive Care Unit of a 1,200-bed tertiary care University Hospital.

## Specimen collection and bacterial isolation:

The bacterial isolates were collected and isolated according to the guidelines. Non-duplicate ESBL-producing *E. coli* isolates were all taken into account in the study period and they were recovered as a result of clinical specimens of all ICU-admitted patients. The clinical specimens included blood cultures, urine, respiratory secretions (endotracheal aspirates and bronchoalveolar lavage), wound swabs, body fluids and catheter tips. Only the original isolate of each patient of each specimen type was selected to prevent multiplication.

## Inclusion criteria:

ESBL-producing *E. coli* isolates obtained in clinical samples of patients staying at the ICU at least 48 hours of the course, which are healthcare-associated infections.

## Exclusion criteria:

Surveillance cultures Isolates of the same patient and type of specimen, polymicrobial cultures *E. coli* was not the predominant pathogen and patients admitted less than 48 hours.

## Bacterial identification:

The identification of bacteria was performed using 2, 4-DNA agar plates, which were confirmed by phenotypic ESBL identification. Isolates were all identified to the species level by standard biochemical methods (indole, methyl red, Voges-Proskauer, citrate utilisation, triple sugar iron agar) and by BD Phoenix automated identification and susceptibility system. The production of ESBL was first screened with cefotaxime and ceftazidime discs (30 µg each) and then confirmed by the combination disc diffusion technique with cefotaxime and ceftazidime alone and in combination with clavulanic acid (10 µg) as suggested by the Clinical and Laboratory Standards Institute (CLSI) 2023 guidelines. The phenotypic ESBL confirmation was ≥5 mm of zone diameter increase of the combination disc over the cephalosporin disc alone. Antimicrobial Susceptibility Testing refers to the practice of evaluating the sensitivity of a specific microorganism to a particular antimicrobial agent to determine effective treatment options. Mueller-Hinton agar on CLSI 2023 standards was used to conduct antimicrobial susceptibility testing based on the Kirby-Bauer disc diffusion technique. Antimicrobial agents were tested as follows: ampicillin (10 µg), amoxicillin-clavulanate (20/10 µg), and piperacillin-tazobactam (100/10 µg) and cefotaxime (30 µg), ceftazidime (30 µg), cefepime (30 µg), aztreonam (30 µg), tobramycin (10 µg), Trimethop. The determination of Colistin minimum inhibitory concentration (MIC) was done through broth microdilution as recommended by the European Committee on Antimicrobial Susceptibility Testing (EUCAST). The quality control strain was *Escherichia coli* ATCC 25922.

## DNA extraction:

Overnight cultures were subjected to the boiling lysis method of genomic DNA extraction. In brief, three to five colonies were resuspended in nuclease-free water, heated at 100°C, 10 minutes, cooled on ice and centrifuged at 14,000 rpm, 5 minutes. The supernatant with the extracted DNA was kept in -20°C. The quality of DNA and concentration was determined by a NanoDrop 2000 spectrophotometer (Thermo Fisher Scientific, Waltham, MA, USA).

## PCR detection of ESBL genes:

Multiplex and simplex PCR were done to identify the key ESBL gene families on the basis of previously tested primer sequences. Amplified gene targets included: blaCTX-M (CTX-M group-specific primers 1, 2, 8, 9 and 25), blaTEM, blaSHV and blaOXA-1-like. The amplification was performed using a thermal cycler (Veriti 96-Well, Applied Biosystems, Waltham, MA, USA) with the following general conditions of the cycling: initial denaturation of 94°C during 5 min; 30 cycles of denaturation at 94°C during 30 s; annealing at the primer-specific temperature during 30 s; extension at 72°C during 60 s; and finally extension at 72°C during 7 min. The products that have been amplified were resolved through electrophoresis using 1.5 percent agarose gels with ethidium bromide and viewed under ultraviolet light. Identification of variants at the level of gene family was done by subjecting representative amplicons of each gene family to bidirectional Sanger sequencing (Macrogen Inc., Seoul, South Korea) protocol. The analysis of sequences was performed with the help of NCBI BLAST tool and the Lahey Clinic beta-lactamase database.

## Determination of phylogenetic group:

Phylogenetic analysis was done based on the revised quadruplex PCR technique on the genes chuA, yjaA, arpA and TspE4.C2 DNA fragment. This technique provides a phylogenetic grouping of the isolates into eight groups, namely, A, B1, B2, C, D, E, F and clade I; the group assignment was made based on the presence or absence of all four genes based on the validated decision algorithm.

## Multilocus sequence typing:

MLST was done based on Achtman scheme, using seven housekeeping genes, adk, fumC, gyrB, icd, mdh, purA and recA. PCR amplification and sequencing of each locus were carried out and allele numbers and sequences types (STs) were obtained with the help of EnteroBase MLST database (https://enterobase.warwick.ac.uk). On shared allelic profiles, clonal complexes were defined by shared profile.

## Statistical analysis:

The SPSS 26.0 (IBM Corp., Armonk, NY, USA) and R 4.3.1 software were used to analyse the data. Frequencies and percentages were used to represent categorical variables. The continuous variables were represented in mean and standard deviation (SD). The chi-square test or Fisher exact test was used to determine associations between variables that are categorical. The diversity index of Simpson was then estimated to determine the genetic diversity of the sequence types. The statistical significance of a p-value was taken as below 0.05. PHYLOViZ version 2.0 was used to create minimum spanning trees on MLST data.

## Results:

During the study period, a total of 186 non-duplicates ESBL-producing *E. coli* isolate meeting the inclusion criteria were recovered from ICU patients. The mean age of affected patients was 54.8 ± 18.6 years, with 112 (60.2%) being male. The medical ICU contributed the highest number of isolates (n=98, 52.7%), followed by the surgical ICU (n=62, 33.3%) and the neonatal ICU (n=26, 14.0%). The most common specimen source was urine (n=72, 38.7%), followed by blood (n=44, 23.7%), respiratory secretions (n=38, 20.4%), wound swabs (n=22, 11.8%) and other body fluids (n=10, 5.4%). PCR and sequencing analysis revealed a diverse repertoire of ESBL genes among the 186 isolates. The blaCTX-M gene family was the most prevalent, detected in 153 isolates (82.3%), followed by blaTEM in 104 (55.9%), blaSHV in 48 (25.8%) and blaOXA-1-like in 62 (33.3%). Among CTX-M-positive isolates, CTX-M group 1 was predominant (n=132, 86.3%), followed by CTX-M group 9 (n=18, 11.8%) and CTX-M group 2 (n=3, 1.9%). Sequencing identified blaCTX-M-15 as the most common variant (n=118, 63.4% of all isolates), followed by blaCTX-M-14 (n=14, 7.5%), blaCTX-M-27 (n=8, 4.3%) and blaCTX-M-55 (n=6, 3.2%). Multiple ESBL gene co-carriage was observed in 78 isolates (41.9%), with the combination of blaCTX-M + blaTEM being the most frequent (n=52, 28.0%). The complete gene distribution data are presented in Table 1. Phylogenetic classification revealed that group B2 was the most prevalent (n=72, 38.7%), followed by group D (n=51, 27.4%), group A (n=28, 15.1%), group B1 (n=18, 9.7%), group F (n=9, 4.8%) and groups C and E (n=4 each, 2.2% each). MLST analysis identified 28 distinct sequence types, yielding a Simpson's diversity index of 0.891. The predominant sequence types were ST131 (n=46, 24.7%), ST405 (n=22, 11.8%), ST648 (n=16, 8.6%), ST38 (n=12, 6.5%), ST410 (n=10, 5.4%) and ST69 (n=8, 4.3%). The remaining 72 isolates (38.7%) were distributed across 22 additional sequence types, each comprising fewer than 5% of isolates. The association between dominant sequence types and phylogenetic groups, ESBL genes and specimen sources is presented in Table 2. All 186 isolates were resistant to ampicillin and showed confirmed ESBL phenotypes against third-generation cephalosporins. High co-resistance rates were observed for trimethoprim-sulfamethoxazole (81.7%), ciprofloxacin (78.5%), levofloxacin (74.2%), tobramycin (61.3%) and gentamicin (54.8%). Carbapenem resistance was detected in 7 isolates (3.8%). Susceptibility was highest for colistin (99.5%), tigecycline (96.2%), fosfomycin (93.0%), amikacin (87.6%) and nitrofurantoin (84.4%). Multidrug resistance (MDR), defined as non-susceptibility to at least one agent in three or more antimicrobial categories, was observed in 164 isolates (88.2%). Extensively drug-resistant (XDR) phenotypes were identified in 14 isolates (7.5%). The complete antimicrobial susceptibility profile is presented in Table 3.

## Discussion:

The present research presents an end-to-end molecular characterisation of ESBL-producing *E. coli* isolates of ICU patients, showing a high degree of genetic diversity and the prevalence of globally recognised high-risk clonal lineages. Their results show the multifaceted interaction between the spread of resistance genes, clonal spread and the individual environment of the ICU. The current prevalence of the blaCTX-M gene family in 82.3% of isolates confirms the global trend shift toward CTX-M enzymes that has been reported to take place since the early 2000s, as a replacement of TEM and SHV-type ESBLs [14]. The prevalence of blaCTX-M-15 with a prevalence rate of 63.4 in all isolates reflects the pandemic spread of this variant of which has been observed in various geographic environments, both in healthcare settings and community environments in sub-Saharan Africa and South Asia [15]. The effectiveness of CTX-M-15 can be explained by the fact that it is often connected with highly transmissible plasmids, especially those that belong to the IncF replicon group and carry several resistance determinants and ensure effective horizontal gene transfer [16]. The high rate of co-carriage with ESBL gene in the current study (41.9) is a very alarming event in as far as the build-up of multiple resistance mechanisms in single isolates is concerned. The presence of blaCTX-M with blaTEM and blaOXA genes indicates the ability of mobile genetic elements to possess multiple beta-lactamase genes as well as antimicrobial resistance mechanisms to other antibiotic classes [17]. Such a co- carriage pattern can be attributed to extremely high rates of multidrug resistance (88.2) and high levels of co-resistance to fluoroquinolones, aminoglycosides and trimethoprim-sulfamethoxazole in this population of the ICU [18]. These profiles of resistant phenotypes have a serious impact on empirical treatment and highlight the relevance of carbapenems as the last-resort drugs, which were also displaying rising resistance in 3.8% of isolates. The fact that most ESBL-producing isolates (38.7%) belonged to the phylogenetic group B2 is an interesting discovery that has significant clinical consequences. The group B2 strains are generally known to be the most virulent extraintestinal pathogenic lineage of *E. coli* with the highest diversity of virulence factors such as adhesins, siderophores, toxin and immune evasion system [19]. The intersection of increased virulence and ESBL-based resistance in these strains is a notably dangerous phenotypic intersection and could be part of more serious clinical outcomes and increased attributable mortality in ICU patients [20]. This is in line with the classification of ST131 (24.7) as the most dominant type of sequence, as it is the most successful pandemic clone of ESBL-producing *E. coli* in the world. This lineage has been found to encode an exceptionally strong repertoire of virulence determinants together with the ability to resist multidrug resistance, achieve effective colonisation of intestines and person- to-person transmission [21]. The high rate of fluoroquinolone resistance (93.5) in ST131 isolates in this study is typical of the H30 subclone which contains chromosomally encoded mutations of the quinolone resistance-determining regions of gyrA and parC genes [22]. The high correlation of ST131 to bloodstream infections in this research also supports the invasive nature of this lineage that was found to be the major contributor to *E. coli* bacteraemia in most healthcare systems [23].

The expansion and maintenance of 28 different types of sequence variants among 186 isolates to give a Simpson index of 0.891 is evidence that ESBL transmission in this ICU environment is contributed to by both clonal expansion of predominant lineages and horizontal gene transfer among genetically diverse isolates [24]. This two-way system of resistance spread creates significant difficulties with regard to infection control. Although theoretically clonal outbreaks may be limited through direct treatment, i.e., patient isolation and hand hygiene, the horizontal transmission of resistance plasmids between different commensal and pathogenic strains is much harder to restrict [25]. Of special interest is the appearance of secondary high-risk clones outside of ST131. The ST405, ST648 and ST410 that contributed 25.8% of isolates in this study have been gaining acceptance as significant vehicles of ESBL and carbapenemase spread in current world surveillance studies [26]. Specifically, ST410 has been reported to be an emerging high-risk clone, which is shown to possess the ability to obtain multiple carbapenemase genes, implying that the development of the highly resistant lineages in the ICU setting should be monitored regularly by molecular means [27]. The fact that carbapenem resistance was observed in 3.8% of ESBL producing isolates is a worrying trend that needs to be addressed immediately. Although the prevalence is quite low, the ICU setting offers the best selection and amplification of carbapenem-resistant subpopulations by prolonged antibiotic pressure [28]. Carbapenem resistance in the ESBL producers could reflect the gain of other resistance mechanisms, such as the production of carbapenemases or loss of porins along with amplification of overexpression of AmpC [29]. Susceptibility rates of colistin (99.5%), tigecycline (96.2%) and fosfomycin (93.0%) are positive because they are the agents of last resort to manage highly resistant infections. Nevertheless, the reported worldwide outbreak of plasmid-mediated colistin resistance with the help of the mcr genes and the growing incidents of fosfomycin resistance require constant watchfulness in keeping track of the susceptibility trends [30]. There are a number of drawbacks of this study that have to be recognized. The single-centre design can restrict the external validity of the results to other healthcare environments with diverse patient populations, antibiotic use and infection control procedures. Cross-sectional design does not allow one to establish the temporal trends or monitor clonal dynamics across time. It did not perform whole-genome sequencing that would offer a more detailed characterisation of mechanisms of resistance and virulence determinants as well as transmission pathways, but this is an essential route to take in future studies [31]. The study has also failed to incorporate environmental or healthcare worker screening, which would have given a good background about possible reservoirs and the avenues of transmission in the ICU [32].

## Conclusion:

ESBL-producing *E. coli* in ICU environments are characterized by impressive genetic diversity with blaCTX-M-15 being the most common resistance determinant that is mostly present in the pandemic high-risk clone ST131 as well as a variety of overlapping clonal lineages and multidrug resistance. The intersection of virulent phylogenetic backgrounds, the co-carriage of ESBL genes and the development of carbapenem resistance in this ICU group is a critical point to the issue of integrated molecular surveillance programmes and the need to address antimicrobial stewardship through specific antimicrobial surveillance and response programmes. This evidence highlights that in ICU settings, both the confinement of predominant clonal lineages and the breakage of horizontal gene transfer of resistance among genetically divergent *E. coli* groups are necessary in order to foster effective infection control practices.

## Figures and Tables

**Table 1:** Distribution of ESBL gene families and variants among 186 isolates

**ESBL Gene**	**n (%)**	**Most Common Variant**	**Variant n (%)**
blaCTX-M (total)	153 (82.3%)	-	-
CTX-M Group 1	132 (71.0%)	blaCTX-M-15	118 (63.4%)
CTX-M Group 9	18 (9.7%)	blaCTX-M-14	14 (7.5%)
CTX-M Group 2	3 (1.6%)	blaCTX-M-2	3 (1.6%)
blaTEM (total)	104 (55.9%)	blaTEM-1 (non-ESBL)	86 (46.2%)
		blaTEM-52	10 (5.4%)
		blaTEM-30	8 (4.3%)
blaSHV (total)	48 (25.8%)	blaSHV-12	22 (11.8%)
		blaSHV-11 (non-ESBL)	16 (8.6%)
		blaSHV-5	10 (5.4%)
blaOXA-1-like	62 (33.3%)	blaOXA-1	58 (31.2%)
Co-carriage patterns			
CTX-M + TEM	52 (28.0%)	-	-
CTX-M + OXA	38 (20.4%)	-	-
CTX-M + TEM + OXA	28 (15.1%)	-	-
CTX-M + SHV	24 (12.9%)	-	-
CTX-M + TEM + SHV + OXA	12 (6.5%)	-	-

**Table 2:** Association between major sequence types, phylogenetic groups and ESBL genes

**Sequence Type**	**n (%)**	**Phylogenetic Group**	**Predominant ESBL Gene**	**Fluoroquinolone Resistance n (%)**	**Specimen Source (Most Common)**
ST131	46 (24.7%)	B2	blaCTX-M-15 (89.1%)	43 (93.5%)	Blood (34.8%), Urine (32.6%)
ST405	22 (11.8%)	D	blaCTX-M-15 (72.7%)	18 (81.8%)	Urine (40.9%), Respiratory (27.3%)
ST648	16 (8.6%)	F	blaCTX-M-15 (62.5%)	14 (87.5%)	Urine (37.5%), Wound (25.0%)
ST38	12 (6.5%)	D	blaCTX-M-14 (50.0%)	7 (58.3%)	Urine (50.0%), Blood (25.0%)
ST410	10 (5.4%)	A	blaCTX-M-15 (80.0%)	8 (80.0%)	Respiratory (40.0%), Blood (30.0%)
ST69	8 (4.3%)	D	blaCTX-M-15 (62.5%)	4 (50.0%)	Urine (62.5%), Other (25.0%)
Others (22 STs)	72 (38.7%)	Mixed	Mixed	52 (72.2%)	Mixed
Chi-square analysis:
significant association between
ST131 and phylogenetic group B2 (p<0.001);
significant association between
ST131 and fluoroquinolone resistance (p=0.003).

**Table 3:** Antimicrobial Susceptibility Profile of 186 ESBL-Producing *E. coli* Isolates

**Antimicrobial Agent**	**Susceptible n (%)**	**Intermediate n (%)**	**Resistant n (%)**
Ampicillin	0 (0%)	0 (0%)	186 (100%)
Amoxicillin-clavulanate	68 (36.6%)	46 (24.7%)	72 (38.7%)
Piperacillin-tazobactam	128 (68.8%)	26 (14.0%)	32 (17.2%)
Cefotaxime	0 (0%)	4 (2.2%)	182 (97.8%)
Ceftazidime	14 (7.5%)	12 (6.5%)	160 (86.0%)
Cefepime	42 (22.6%)	28 (15.1%)	116 (62.4%)
Aztreonam	18 (9.7%)	16 (8.6%)	152 (81.7%)
Imipenem	178 (95.7%)	2 (1.1%)	6 (3.2%)
Meropenem	179 (96.2%)	1 (0.5%)	6 (3.2%)
Ertapenem	176 (94.6%)	3 (1.6%)	7 (3.8%)
Ciprofloxacin	38 (20.4%)	2 (1.1%)	146 (78.5%)
Levofloxacin	44 (23.7%)	4 (2.2%)	138 (74.2%)
Gentamicin	80 (43.0%)	4 (2.2%)	102 (54.8%)
Amikacin	163 (87.6%)	8 (4.3%)	15 (8.1%)
Tobramycin	66 (35.5%)	6 (3.2%)	114 (61.3%)
Trimethoprim-sulfamethoxazole	32 (17.2%)	2 (1.1%)	152 (81.7%)
Nitrofurantoin	157 (84.4%)	12 (6.5%)	17 (9.1%)
Fosfomycin	173 (93.0%)	4 (2.2%)	9 (4.8%)
Tigecycline	179 (96.2%)	4 (2.2%)	3 (1.6%)
Colistin	185 (99.5%)	0 (0%)	1 (0.5%)
MDR isolates:
164/186 (88.2%);
XDR isolates:
14/186 (7.5%).
